# Immunosuppressive Effects of Thallium Toxicity in Nile Tilapia Fingerlings: Elucidating the Rescue Role of *Astragalus membranaceus* Polysaccharides

**DOI:** 10.3389/fvets.2022.843031

**Published:** 2022-06-09

**Authors:** Mayada R. Farag, Mahmoud Alagawany, Samah R. Khalil, Eman W. El-Hady, Walaa M. Elhady, Tamer Ahmed Ismail, Carlotta Marini, Alessandro Di Cerbo, Hany M. R. Abdel-Latif

**Affiliations:** ^1^Forensic Medicine and Toxicology Department, Faculty of Veterinary Medicine, Zagazig University, Zagazig, Egypt; ^2^Poultry Department, Agriculture Faculty, Zagazig University, Zagazig, Egypt; ^3^Department of Clinical Laboratory Sciences, Turabah University College, Taif University, Taif, Saudi Arabia; ^4^School of Biosciences and Veterinary Medicine, University of Camerino, Matelica, Italy; ^5^Department of Poultry and Fish Diseases, Faculty of Veterinary Medicine, Alexandria University, Alexandria, Egypt

**Keywords:** toxicity, immunity, hematology, attenuation, herbal medicines, diseases

## Abstract

This study evaluated the immunotoxic effects of thallium (Tl) in Nile tilapia fingerlings and the recovery role of dietary *Astragalus membranaceus* polysaccharides (ASs). An 8-week experiment was designed where 180 fishes were randomly and equally assigned in triplicates into the six groups: the control group (CNT) was reared in unpolluted water and fed a commercial diet, two groups were fed a well-balanced commercial diet plus 1.5 and 3.0 g AS/kg diet (AS0.15 and AS0.30), respectively, the fourth group was exposed to a sublethal dose of Tl (41.9 μg l^−1^) [equal to 1/10 of 96-h lethal concentration 50 (LC50)], and the last two groups were fed 0.15 and 0.3% AS, respectively, and concurrently exposed to Tl (41.9 μg l^−1^) (AS0.15+Tl and AS0.30+Tl). Fish hematobiochemical parameters, serum immunity [nitric oxide, total immunoglobulin M (IgM) levels, and lysozyme activity], transcription of hepatic interferon-γ (IFN-γ), interleukin-1β (IL-1β), and tumor necrosis factor-α (TNF-α), and resistance to *Aeromonas hydrophila* (*A. hydrophila*) were assessed. Hematobiochemical parameters and serum immune indices were significantly decreased in the fish group exposed to sublethal Tl concentration compared to the CNT group. Furthermore, Tl exposure significantly induced overexpression of IL-1β, TNF-α, and IFN-γ genes (4.22-, 5.45-, and 4.57-fold higher, respectively) compared to CNT values. Tl exposure also increased the cumulative mortality (%) in Nile tilapia challenged with *A. hydrophila*. Remarkably, the groups fed AS0.15+Tl and AS0.30+Tl significantly ameliorated all the aforementioned parameters, but did not reach CNT values. Our findings suggest the possible immunomodulating roles of dietary AS in recovering the immunotoxic effects of Tl in Nile tilapia. We can conclude that dietary AS would be useful for maintaining the immunity of Nile tilapia fingerlings.

## Introduction

Thallium (Tl) is a rare-earth element and is regarded as an extremely toxic heavy metal (1). Tl can occur in two forms: monovalent Tl(I) and trivalent Tl(III) forms (2). Currently, Tl gained particular interest among numerous trace metals due to its attractive applications as a product obtained from the mining of coal, glass manufacture, electric materials, and several insecticides (3). Nonetheless, the unhygienic release of Tl over large geographic areas led to increased pollution of the aquatic ecosystems, including water, bottom sediments, and aquatic organisms. The primary Tl sources are closely related to effluents from coal-based industries (processing and mining of coal and coal-firing plants) and refining sulfide-rich natural rocks or sediments (4). As a result, it can be considered an apparent environmental contaminant to aquatic environments.

Several literature reports elucidated that monovalent Tl toxicity in aquatic organisms inhabiting the freshwater environments ranged between μg/l and mg/l (5–11). Importantly, it is crucial to determine the levels of Tl able to result toxic in the exposed organisms. It was found that Tl level ranging between 1 and 60 ppm that slowly killed fish (12). Moreover, the 72-h lethal concentration 50 (LC50) of Tl is 10–15 mg l^−1^ for *Oncorhynchus mykiss*, 0.03 mg l^−1^ for *Salmo salar*, 2–4 mg l^−1^ for invertebrates and aquatic insects, and 0.4 mg l^−1^ for tadpoles. Likewise, the 96-h LC50 for *Danio rerio* (*D. rerio*) was 870 μg l^−1^ (13).

Hou et al. observed that the exposure of *D. rerio* to environmentally relevant concentrations of Tl considerably increased the expression of heat shock protein 70 (HSP70) and metallothionein 2 (Mt2) genes, elevated superoxide dismutase activity in the liver tissue, and increased the Na^+^/K^+^-ATPase in the gill tissues (14). The same authors also found that Tl exposure induced severe histopathological changes in gonads, gills, and liver tissues. Further, Li et al. illustrated that exposure of *D. rerio* to 0.1 μg l^−1^ Tl (I) for 15 consecutive days produced physiological alterations in ammonia nitrogen excretion, oxygen consumption rate, and ammonia quotient, indicating rapid increase in protein metabolism and anaerobic energy utilization (15).

Among the wide range of Chinese herbs usually used as traditional medicines, *Astragalus membranaceus* (AM) is considered to be endowed with immunomodulatory, hepatoprotective, and antioxidant activities (16, 17). Its principal functional bioactive compounds are polysaccharides (PSs), flavonoids, phenolic acids, alkaloids, and saponins (18). PSs are the most crucial bioactive compounds in AM with numerous pharmacological advantages (19, 20). Reports have proven that injection of *Astragalus membranaceus* polysaccharides (ASs) considerably increased the transcription of immune-related genes in *Cyprinus carpio* (*C. carpio*) (21). Furthermore, Sun et al. showed that AS noticeably enhanced the immune responses of sea cucumber (22).

Dietary AS enhanced the growth parameters (initial and final weight, weight gain, feed intake, specific growth rate, and feed conversion ratio) and immune parameters (phagocytic, respiratory burst, and bactericidal and plasma lysozyme activities) of Nile tilapia (*Oreochromis niloticus*) (23), modulated the gut microbiome of sea cucumber (24, 25), and increased the immunity (phagocytic, respiratory burst, lysozyme and inducible nitric oxide synthase, and content activity) of largemouth bass, enhancing their resistance to *Aeromonas hydrophila* (*A. hydrophila*) (26). Furthermore, dietary AS improved the antioxidant status [superoxide dismutase (SOD), glutathione peroxidase, and total antioxidant capacity] of large yellow croaker and turbot (23, 27), enhancing the resistance of juvenile crucian carps (28) and grass carps (29) to *A. hydrophila*, as well as the antiviral immunity of zebrafish (30).

Several attempts to find natural and safe feed supplements to attenuate the toxicity signs in fishe have been proposed so far. In this sense, it was found that supplementing diets with AS also induced hepatoprotection and increased the antioxidant status of *C. carpio* exposed to carbon tetrachloride (CCl_4_) (31). AM has also been regarded as an important natural antistress feed additive for *Perca flavescens* (32) and *Lepomis macrochirus* (33). Notably, AM extract powder increased the survivability of Nile tilapia exposed to low-temperature stress (34). Our recently published study elucidated that AS could enhance the growth, hematobiochemical indices (alanine aminotransferase, creatinine, total cholesterol, triglycerides, glucose, and cortisol), hepatic antioxidant status [catalase, SOD enzyme activity, and malondialdehyde (MDA) content], and transcription of apoptosis-related and Hsp70 genes in Nile tilapia fingerlings exposed to sublethal Tl toxicity (35).

Herein, we have assessed the potential effectiveness of dietary AS in the mitigation of hematobiochemical and immune indices, transcription of immune related, and disease resistance of Nile tilapia exposed to sublethal Tl toxicity.

## Materials and Methods

### Thallium and *Astragalus membranaceus* Polysaccharide

Thallium (I) nitrate (99.9% purity) was obtained from Sigma-Aldrich (St. Louis, Mosby, USA; CAS Number: 10102-45-1). AS (60% purity) was obtained from the local market (El-Ahmadeya Company, Egypt). The analysis showed that AS was composed of arabinose, α-1,4-glucan, α-1,6-glucan, galacturonic acid, rhamnose, and galactose. Analytical grade chemicals and reagents were utilized in this study.

### Animals and Housing

Nile tilapia fingerlings (body weight = 13 g ± 1.00) were purchased from a fish hatchery (Abbassa, Egypt). Fishes were adapted for 15 days in 500 L rearing tanks. During the adaptation period, fisher were fed control (basal) well-balanced diet (Aller Aqua Company, Egypt). This diet contains all the nutritional requirements for better growth of Nile tilapia (36). Fisher were fed three times daily (5% of their biomass). The ingredients and constituents of ration and its chemical composition are given in Table 1.

**Table 1:** Ration constituents (g kg^−1^ diet) and proximate composition analysis of the control diet (35, 55) used in the experiment.

**Ingredients**	**Amount (g/kg diet)**
Soybean meal (SBM; 48% crude protein)	200
Yellow corn	210
Fish meal (FM)	150
Wheat middlings	150
Corn gluten (CG; 60% crude protein)	130
Rice bran	110
Corn oil	30
Mineral premix^a^	10
Vitamin premix^b^	10
Total	1,000
**Composition analysis**
Crude protein (CP; %)	320.5
Crude lipids (CL; %)	45.50
Crude fiber (CF; %)	42.45
Ash (%)	73.01
Nitrogen free extract (NFE)^c^	518.54

^a^* Mineral premix (Kg^−1^): Manganese (53 g), Zinc (40 g), Iron (20 g), Copper (2.7 g), Iodine (0.34 g), Selenium (70 mg), Cobalt (70 mg), and Calcium carbonate (as carrier) up to 1 kg*.

^b^* Vitamin premix (Kg^−1^): Vitamin C (500 mg), Vitamin A (8.000.000 IU), Vitamin D3 (2.000.000 IU), Vitamin E (7.000 mg), Vitamin K3 (1.500 mg), Vitamin B1 (700 mg), Vitamin B2 (3.500 mg), Vitamin B6 (1.000 mg), Vitamin B12 (7 mg), Biotin (50 mg), Folic acid (700 mg), Nicotinic (20.000 mg), and Pantothenic acid (7.000 mg)*.

*^c^ NFE was calculated as 100 – (CP + CL + ash + CF)*.

During the adaptation period, physical and chemical measurements of the rearing water were 26.5–28.5°C for temperature, 6.50–7.5 mg l^−1^ for dissolved oxygen, 7.45–7.6 for pH, 0.01–0.02 mg l^−1^ for nitrite, 0.01–0.03 mg l^−1^ for nitrates, and 0.01–0.02 mg l^−1^ for unionized ammonia. The photoperiod was stable as 12/12 h as light and dark cycle.

### Diets and Experimental Design

Two different inclusion percentages of AS were used with the ingredients of the control diet 1.5 and 3.0 g AS/kg diet and defined as AS0.15 and AS0.30, respectively. All the constituents were pelletized to form 1.5 mm pellets using carboxymethylcellulose (used in fish nutrition as a pelleting agent), allowed to dry in the air for 1 day at 25°C, and then stored in plastic bags at 4°C in the refrigerator. On the other hand, the 96-h LC50 of thallium (Tl) in Nile tilapia fingerlings used during this study was previously calculated as 419 μg l^−1^ in our recently published study (35).

One hundred eighty fishes were randomly and equally assigned in triplicate to the six experimental groups (*n* = 30); each group was reared in a glass aquarium (sized 80 × 80 × 100 cm) containing 90 L dechlorinated water. The control group (CNT) was reared in an aquarium filled with clean unpolluted water and fed the basal diet only. The AS0.15 and AS0.30 groups were reared in clean water and fed basal diet supplemented with 0.15 or 0.30% AS. The group exposed to sublethal Tl concentration was fed the basal diet and exposed to 1/10th of the 96-h LC50 of Tl (41.9 μg l^−1^) and Tl was dispersed and completely dissolved in deionized water to prevent further toxicity to exposed fishes. The last two groups were fed the basal diet supplemented with 0.15 and 0.30% AS and concurrently exposed to Tl (41.9 μg l^−1^) and were defined as AS0.15+Tl and AS0.30+Tl, respectively. To prevent Tl degradation in the rearing water and keep the exposure level constant, Tl was replenished every 96-h interval. The whole experiment lasted for 8 weeks.

### Sampling Procedures (Blood and Liver Tissues)

All the fishes were kept fasted 1 day prior to sampling. After this, they were anesthetized by MS-222 (tricaine methanesulfonate) in a dose rate of 200 mg l^−1^ to decrease the handling stress (37). Seven fishes per replicate (21 fishes per group) were sampled. Blood was sampled from the fish's caudal vessels (about 1 ml per fish and pooled from three fishes). Blood samples were separated into two parts: three pooled samples mixed with sodium citrate into Eppendorf tubes and used for the hematobiochemical analyses, while the other four pooled samples were left at room temperature and then centrifuged at 3,000 × g for 20 min to collect serum for immunological indices. Serum samples were then stored at −20°C. Liver specimens (100 mg per fish) were sampled after the aseptic opening of the fishes and then quickly frozen in a liquid nitrogen tank. Liver samples were then stored at −80°C for quantitative reverse transcription-PCR (qRT-PCR) analysis.

### Hematobiochemical Parameters

Red blood cells (RBCs) and white blood cells (WBCs) were counted by an automatic cell counter (Hospitex Hema Screen 18, Italy). Values of hemoglobin (Hb) and packed cell volume (PCV) were estimated according to (38). The mean corpuscular volume (MCV) was measured according to (39). Total protein (TP) and albumin (ALB) contents were quantified by using the BioMed Diagnostic Colorimetric Kits (Egy Chem for Lab Technology, Egypt) (40, 41). Globulin (GLO) values were calculated by the difference between TP and ALB values.

### Immunological Assays

Serum nitric oxide (NO) was evaluated spectrophotometrically using commercial kits (BioChain, Incorporation, USA) following the manufacturer's instructions. Serum myeloperoxidase (MPO) activity was assayed according to the method proposed by Kumari and Sahoo (42). Serum total immunoglobulin M (IgM) was determined by using ELISA kits specific for fish (MyBioSource Corporation, San Diego, USA; Cat. No. MBS035038) (43, 44). Serum lysozyme (LYZ) activity was assessed by the turbidimetric assay using lyophilized *Micrococcus lysodeikticus* cells (Sigma-Aldrich, St. Louis, Mosby, USA) as a substrate (45). One LYZ activity unit was defined as the decrease of 0.001 of OD_520_ min^−1^.

### Gene Transcription

Ribonucleic acid extraction was done from 50 mg of the collected liver tissues (100 mg) using TRIzol Reagent (iNtRON Biotechnology, South Korea). The purity and concentration of the extracted RNA were assessed by using a ND-2000 spectrophotometer (NanoDrop 2000, Wilmington, Delaware, USA). 3 μg (0.15 μg/μl) RNA was reverse-transcribed. Complementary DNA (cDNA) synthesis was done by using reverse transcription kits (Quantitect^®^, Qiagen, Germany). The steps displayed during the processes of RNA extraction and cDNA synthesis were achieved in accordance with the directions obtained from the manufacturer. The primer sequences (forward and reverse sense) and the National Center for Biotechnology Information (NCBI) GenBank accession numbers of interleukin-1β (IL-1β), interferon-γ (INF-γ), tumor necrosis factor-α (TNF-α), and β-actin (β-actin) are given in Table 2.

**Table 2:** Primers sequences used for real-time quantitative PCR (qPCR) analysis.

**Genes**	**Forward (5^′^-3^′^) Reverse (5^**′**^-3^**′**^)**	**NCBI GenBank Accession No**.
IL-1β	F: CAAGGATGACGACAAGCCAACC R: AGCGGACAGACATGAGAGTGC	XM_003460625.2
IFN-γ	F: AGCACAACGTAGCTTTCCCT R: TAAACAGGGCAAACAGGTCA	XM_003460533.2
TNF-α	F: CAGGATCTGGCGCTACTCAG R: TAGCTGGTTGGTTTCCGTCC	AY428948.1
β-actin	F: CAGGATGCAGAAGGAGATCACA R: CGATCCAGACGGAGTATTTACG	KJ126772.1

*IL-1β, Interleukin 1beta; IFN-γ, Interferon gamma; TNF-α, Tumor necrosis factor alpha; β-actin, Beta actin*.

The qRT-PCR analysis was done by using a Rotor-Gene Q instrument and the QuantiTect^®^ SYBR^®^ Green PCR Kit (Qiagen, Germany). The thermocycling conditions were determined as 95°C for 10 min, followed by 40 cycles of 95°C for 15 s and 60°C for 30 s and 72°C for 30 s. A melt-curve analysis was done to confirm the specificity of PCR products. The relative expression profile of each target gene to β-actin (reference gene) was assessed using the 2-^ΔΔ*Ct*^ method (46).

### Challenge Test

*Aeromonas hydrophila* used in the challenge test was previously isolated from diseased Nile tilapia and identified in our previous study (47). The bacterium was cultured in Tryptic Soy Broth (TSB) (Merck, Germany) at 28°C for 24 h (48). The bacterial suspension was then centrifuged at 5,000 × g for 5 min at 25°C. The supernatant was discarded and the pellets were washed 3 times using sterile phosphate-buffered saline (PBS) and then resuspended into the same solution and adjusted to McFarland Standard No. 0.5 at a wavelength of 600 nm, which corresponds to a concentration of 1.5 × 10^8^ cells ml^−1^. The bacterial concentration of 10^8^ cells ml^−1^ was previously reported as sufficient to kill more than half of the challenged Nile tilapia (49).

Another set of the experimental groups (30 fishes/group) were received an intraperitoneal (IP) injection of 0.1 ml of cell suspension containing 1.5 × 10^7^ cells ml^−1^ (50). The mortalities were daily recorded for 2 weeks following the challenge. The relative percentage survival (RPS) and cumulative mortality rate (CMR) of fishes were calculated according to previously published formulas (51).


$$\begin{array}{l} \text{RPS }(\%)=(\text{1}-\text{Mortality in the experimental groups/}\\ \text{ Mortality in the CNT group})\;\times \;100\\ \text{CMR }(\%)=(\text{Total number of dead fishes after injection/}\\ \text{ Number of injected fishes})\;\times \;100 \end{array}$$

### Statistical Analysis

Results were statistically analyzed by one-way ANOVA using SPSS (SPSS version 16.0, SPSS Incorporation, USA). The Tukey's multiple comparisons were used as *post-hoc* to compare means between the groups. The statistical significances were approved when *P* < 0.05. Data were expressed as means ± SEM.

## Results

### Hematobiochemical Parameters

Erythrogram variables such as RBCs count and PCV values exhibited no significant differences among all the experimental groups. However, Tl exposure caused a significant decline (*P* < 0.05) in Hb content and increase in MCV value compared with the CNT group. Dietary supplementation with AS in both the AS0.15+Tl and AS0.30+Tl groups induced a significant modulation in Hb and MCV values compared with the fish group exposed to sublethal Tl concentration, but not completely restored to the CNT values (Table 3).

**Table 3:** Effect of dietary supplementation with *Astragalus membranaceus* polysaccharides (AS) levels (0.15 and 0.30%) and/or exposure to sublethal thallium (Tl) toxicity [1/10th lethal concentration 50 (LC50); 41.9 μg/l] for 8 weeks on the hematological variables and blood protein profile of Nile tilapia.

**Parameters**	**Experimental groups**					
	**CNT**	**AS1.5**	**AS0.30**	**Tl**	**AS0.15+Tl**	**AS0.30+Tl**
**Hematological variables**						
Hb (g/dl)	7.30, 0.34^a^	7.70, 0.35^a^	7.72, 0.86^a^	3.06, 0.38^b^	5.35, 0.08^c^	5.55, 0.19^c^
RBCs (10^6^/μL)	2.68, 0.72	2.53, 1.04	2.80, 0.86	2.00, 0.36	1.73, 0.36	1.80, 0.38
PCV (%)	25.07, 5.00	25.16, 5.07	25.35, 5.25	20.42, 0.10	22.24, 0.46	22.50, 0.37
MCV (fl)	116.38, 1.96^c^	111.54, 0.89^d^	110.24, 0.58^d^	148.05, 1.67^a^	131.15, 0.52^b^	130.81, 0.23^b^
WBCs (10^3^/ mm^3^)	4.99, 0.06^b^	5.04, 0.15^b^	5.29, 0.06^b^	3.68, 0.34^a^	4.69, 0.11^b^	4.81, 0.13^b^
**Blood proteins**						
Total protein (g/dL)	4.24, 0.05^a^	4.36, 0.23^a^	4.46, 0.44^a^	2.65, 0.21^b^	2.33, 0.20^b^	4.16, 0.13^a^
Albumin (g/dL)	1.54, 0.12^a^	1.58, 0.12^a^	1.96, 0.09^a^	0.95, 0.04^b^	0.93, 0.03^b^	1.48, 0.24^a^
Globulin (g/dL)	2.70, 0.11^a^	2.78, 0.35^a^	2.50, 0.39^a^	1.70, 0.17^ab^	1.40, 0.18^b^	2.69, 0.17^a^

*Values are mean ± SEM, values are not sharing a common superscript letter (a, b, c, & d) differ significantly at P <0.05*.

When compared with the values of the CNT group, Tl exposure elicited a significant decline (*P* < 0.05) in the WBCs count (Table 3). In both the AS0.15+Tl and AS0.30+Tl groups, there were significant improvements in WBCs counts that attained the CNT values.

Total protein, ALB, and GLO values were significantly decreased the Tl-exposed group in comparison to the CNT levels. Dietary supplementation with AS in the AS0.30+Tl group induced complete restoration of these values to be non-significantly differed from the CNT values.

### Immunological Indexes

**Figure 1:**
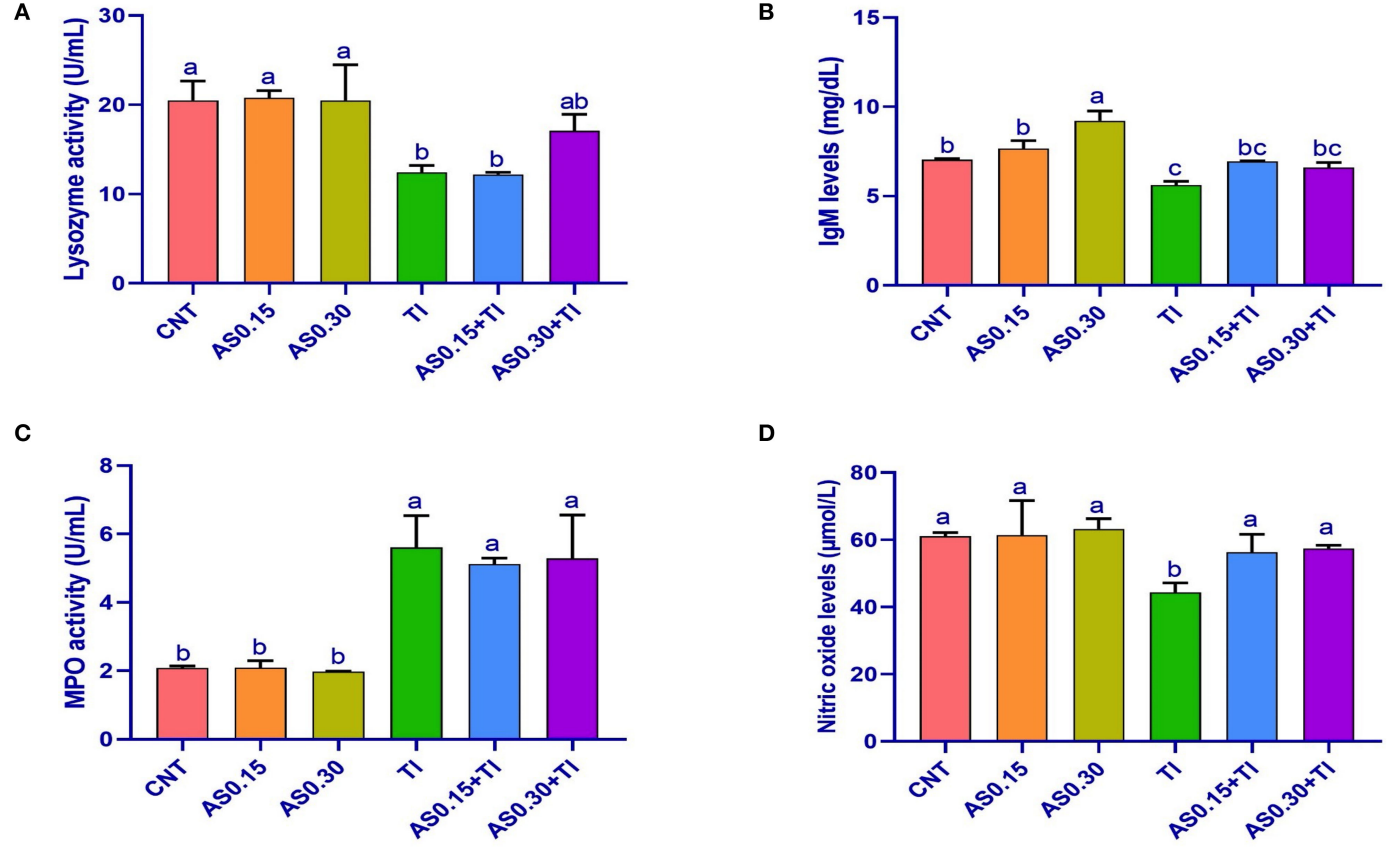
Serum lysozyme activity **(A)**, immunoglobulin M (IgM) levels **(B)**, myeloperoxidase activity **(C)**, and nitric oxide levels **(D)** in Nile tilapia fed diets supplemented with *Astragalus membranaceus* polysaccharides (AS) levels (0.15 and 0.30%) and/or exposed to sublethal thallium (Tl) toxicity [1/10th lethal concentration 50 (LC50); 41.9 μg/l] for 8 weeks. Values are mean ± SE and bars not sharing a common superscript letter (a, b, c, and d) differ significantly at *P* < 0.05.

Sublethal Tl toxicity showed an alteration of the immunity indexes, including LYZ, IgM, MPO, and NO values (Figure 1). On the other hand, the AS supplemented groups elicited no differences from the CNT group, except a significant enhancement of IgM content was recorded the AS0.30 group. Serum IgM and NO contents were significantly improved the AS0.15+Tl and AS0.30+Tl groups, where they were completely restored to the CNT levels. Serum LYZ activity was restored in the AS0.30+Tl group to reach the CNT levels (Figure 1). On the other hand, the altered MPO levels recorded in the Tl-exposed group were not improved by AS supplementation (Figure 1).

### Gene Transcription Results

**Figure 2:**
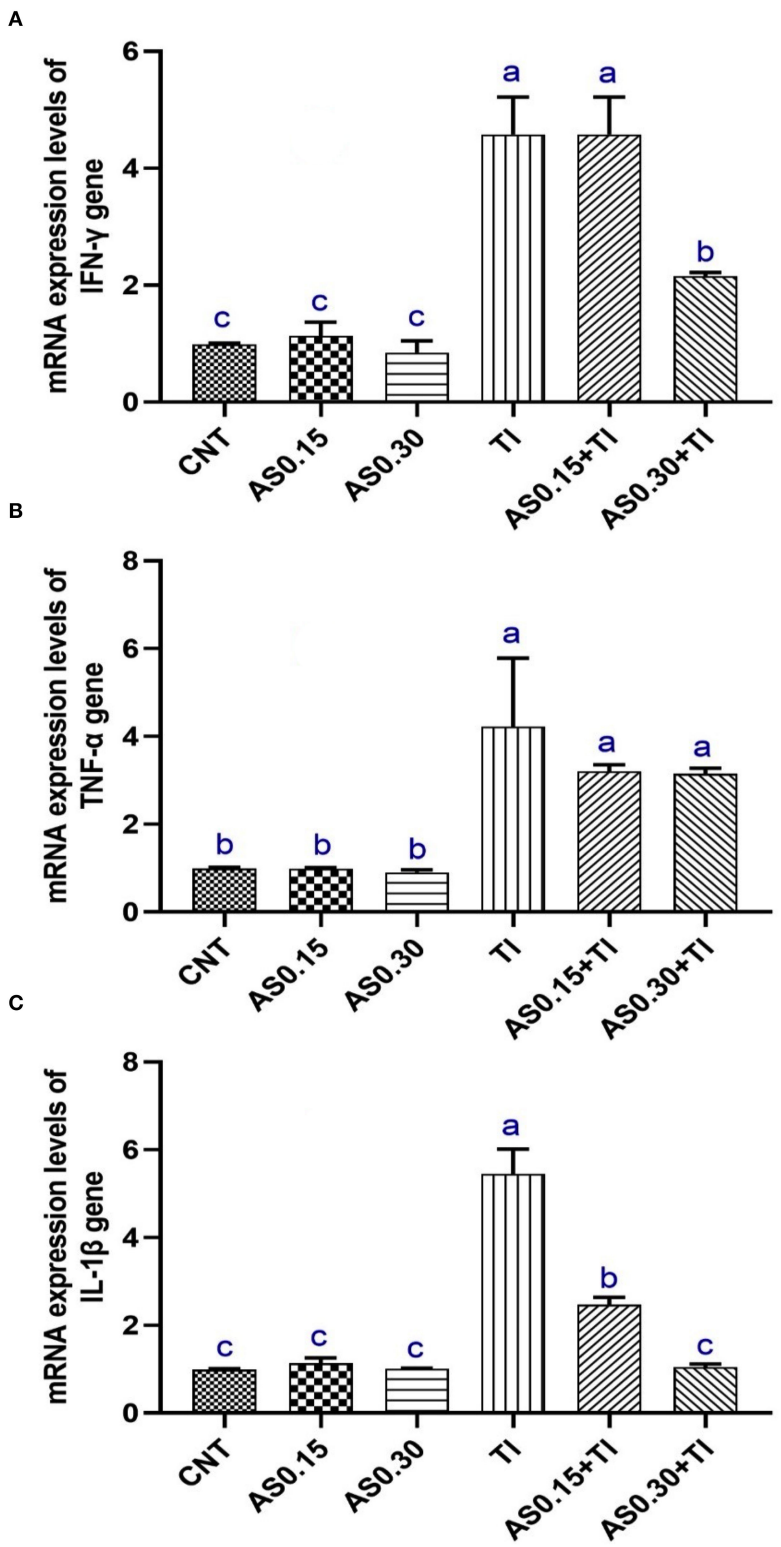
Messenger RNA (mRNA) expression pattern of interferon-γ (INF-γ) **(A)**, tumor necrosis factor-α (TNF-α) **(B)**, and interleukin-1β (IL-1β) **(C)** in liver of Nile tilapia fed diets supplemented with *Astragalus membranaceus* polysaccharides (AS) levels (0.15 and 0.30%) and/or exposed to sub-lethal thallium (Tl) toxicity (1/10th LC50; 41.9 μg/l) for 8 weeks. Values are mean ± SE and bars not sharing a common superscript letter (a, b, c, and d) differ significantly at *P* < 0.05.

Thallium exposure revealed significant overexpression of *IL-1*β, *TNF-*α, and *IFN-*γ genes to be 4.22-, 5.45-, and 4.57-fold higher than the CNT values, respectively (Figure 2). The observed overexpression of *TNF-*α was not modulated by AS in the AS0.15+Tl and AS0.30+Tl groups and showed no significant differences compared with the group exposed to sublethal Tl concentration. Alternatively, a significant modulation was observed in the expression pattern of *IL-1*β in the AS0.15+Tl and AS0.30+Tl groups, particularly in the AS0.30+Tl group as it was completely recovered to attain the normal CNT range. As well, the expression of the *IFN-*γ gene was not mitigated in the AS0.15+Tl group, while modulated only in the AS0.30+Tl group, but did not attain the normal CNT range (Figure 2).

### Fish Resistance Against *Aeromonas hydrophila*

The highest CMR (%) after challenge with pathogenic *A. hydrophila* was observed in the Tl-exposed group (32.33%) followed by the AS0.15+Tl, AS0.30+Tl, and CNT groups (13.33, 10.0, and 6.66%, respectively). On the other hand, no mortalities were recorded in the AS0.15 and AS0.30 groups (Table 4). The challenged fishes exhibited various degrees of escape reflex and abnormal swimming movements. Furthermore, Tl-exposed fishes showed severe signs of infection such as hemorrhagic patches on the abdomen with focal hemorrhage and some ulcerations. The severity of these symptoms was alleviated in the groups whose diets were supplemented with AS.

**Table 4:** Effect of dietary supplementation with *Astragalus membranaceus* polysaccharides (AS) levels (0.15 and 0.30%) and/or exposure to thallium (Tl) toxicity (1/10th LC50; 41.9 μg/l) for 8 weeks on the disease resistance of Nile tilapia challenged with *Aeromonas hydrophila*.

**Parameters**	**Experimental groups**					
	**CNT**	**AS0.15**	**AS0.30**	**Tl**	**AS0.15+Tl**	**AS0.30+Tl**
Total No. of challenged fish	30	30	30	30	30	30
Number of dead fish	2	0	0	7	4	3
Relative percent survival (RPS) (%)	93.33	100	100	76.66	86.66	90
Cumulative mortality rate (CMR) (%)	6.66	0	0	32.33	13.33	10.0

## Discussion

Fish hematology is an important tool for monitoring the fish heath condition and biomarkers for diagnosing fish toxicity (52). In this study, Tl exposure significantly decreased Hb content and increased MCV values. These anemic signs may be due to Tl-induced oxidative stress (14), which is characterized by overproduction of reactive oxygen species (ROS) and a consequent negative impact on Hb concentration (53). In addition, Tl exposure significantly decreased the WBCs, which probably elucidate the immunosuppressant effects of Tl exposure. These effects occur due to the toxic effects of Tl on blood constituents. Similar findings were described after fishes' exposure to pollutants (54, 55).

Notably, dietary supplementation with AS in both the AS0.15+Tl and AS0.30+Tl groups partially restored Tl-induced toxicity on the hematobiochemical parameters. It is well known that herbal extracts have a significant number of polyphenols, which might enrich the cells with iron that has a crucial function in improving the functions of RBCs (56, 57). A possible interpretation of the Hb concentrations improvement may result from the antioxidant effect of AS, which could stabilize Hb level and protect the cell membrane of erythrocytes from the oxidative damage of Tl. Moreover, the improvement of WBCs may be due to the preservation of the leukocyte redox state and the immunomodulatory impact of dietary AS, which may increase the proliferation of leukocytes (55). However, the actual mechanisms involved in enhancement of hematobiochemical parameters of fishes fed dietary AS are still unclear to date.

Blood proteins are bioindicators of the nutritional, immunological, and health conditions of fishes. Moreover, they are principally involved during the exposure of fishes to stressors, as these animals require more energy to overwhelm the effects of these stressors (58). Tl exposure significantly decreased TP, ALB, and GLO levels compared to the CNT group. These findings may be attributed to hepatorenal damage after Tl exposure, which may subsequently increase the vascular leakage and worsen renal glomerular filtration or impair liver functions, which ultimately lead to the inability of the liver to synthesize ALB. On the other hand, TP, ALB, and GLO levels were increased in the AS-supplemented groups. In a similar sense, Jia et al. reported that dietary supplementation with AS (1.5 and 3 g/kg) considerably increased the reduced serum TP and ALB values in CCl_4_-exposed common carps (31). Also, dietary AS increased serum TP and ALB in large yellow croakers (59). Increased serum proteins are closely related to the enhanced immunity by AS feeding.

Lysozyme, MPO, total IgM, and NO are from the immune parameters of fish (60–62). In this study, the fish exposure to sub-lethal Tl toxicity caused a marked decrease in serum LYZ activity and NO and IgM levels in the exposed Nile tilapia, indicating immunosuppressive effects of Tl. Similar findings were also reported after exposure of Nile tilapia to T1 (35) and deltamethrin (35, 63). A recent literature also reported that exposure of fishes to some aquatic toxicants induced immunotoxicity (64).

On the other hand, dietary AS can modulate the immune parameters in the AS0.15+Tl and AS0.30+Tl groups. Dietary AM can also increase the total immunoglobulins and serum LYZ activities in Nile tilapia (65). Plasma LYZ activity was also elevated in Nile tilapia fishes fed AS-supplemented diets (23). Increased serum LYZ activity was also noticed in sea cucumber-based diets supplemented with AS or AM (25). Furthermore, dietary AS enhanced NO production, increased the phagocytic activity of head kidney macrophages and serum LYZ activities in large yellow croakers (59).

Fish cytokines are molecules involved in initiating and regulating the innate immune responses (66, 67). Tumor necrosis factors (TNFs) are implicated in the inflammation process, apoptosis, and cell proliferation (68). Moreover, TNF-α is the most immunologically significant molecule in the TNF family (66). The pro-inflammatory cytokines such as IL-1β, TNF-α, and IFN-γ are from fish cytokines with pivotal immune functions (69–71).

The pro-inflammatory cytokines are molecules that initiate and regulate the fish innate immunity and their increase is indicative of an inflammatory process (72–74). TNF-α is involved in cell proliferation inflammation apoptosis (66) via promoting the release of other cytokines, activating lymphocytes and neutrophils, increasing the endothelial cells permeability, and controlling the metabolic activities of cells (71). Similar functions were reported for IL-1β in fish, where it can regulate the immune and inflammatory responses (75). INF-γ is a key mediator in enhancing MHC-I and II histocompatibility complexes expressions and is involved in the fish immune response (70). In this study, the expressions of IFN-γ, IL-1β, and TNF-α genes were upregulated in liver of Nile tilapia response to sublethal T1 exposure. Similar results were observed in brain tissue of Nile tilapia exposed to sublethal T1. These genes were also upregulated in response to other contaminants associated with inflammation and immunotoxicity such as clethodim in zebrafish embryos (76) and bifenthrin in zebrafish larvae (77, 78) and Nile tilapia (35).

Reports showed that AS could activate the proliferation of B cells and macrophages and increase cytokine production (18). The effects of AS on the expression of cytokines may be linked to the ability of AS in the modulation of the host immunity via Toll-like receptor 4 (TLR4)-mediated myeloid differentiation factor 88 (MyD88)-dependent signaling pathway (79). Moreover, Sun et al. proposed that dietary AS is involved in regulating the hepatic immune responses in turbot via TLRs/nuclear factor-kappa B (NF-κB) signaling pathway (27). We suggest conducting future study and several experiments to elucidate the actual mechanisms of dietary AS in attenuation of the overexpressed genes after sub-lethal Tl toxicity.

The increased survival rates after challenge with pathogenic *A. hydrophila* were observed in the AS-supplemented groups compared with Tl-exposed fishes. The immune-suppressive effects after Tl exposure such as leukocytopenia, hypoproteinemia, decreased serum LYZ, NO, and total IgM will also reduce the resistance of fishes against challenged pathogens; therefore, it will increase the mortalities after challenge with *A. hydrophila*. On the other hand, dietary AS improved the disease resistance of the treated fishes. Dietary AM reduced the mortality rates of Nile tilapia after the challenge with *A. hydrophila* (65). Moreover, Wang et al. reported that supplementing diets with AM or AS considerably lowered the CMR% in sea cucumber challenged with *Vibrio splendidus* (25). Besides, Lin et al. also indicated that dietary AS decreased mortalities of largemouth bass challenged with *A. hydrophila* compared with the CNT fed the basal diet (26). Recently, Shi et al. showed that dietary AS increased the protection of grass carp tissues after a challenge with *A. hydrophila* (29). These effects may be attributed to the powerful antimicrobial effects of AS (18) and the immunomodulatory effects of AM (80).

## Conclusion

Sublethal thallium (Tl) toxicity induced immunosuppression in the exposed Nile tilapia fingerlings. It induced leukocytopenia, hypoproteinemia, and hypoalbuminemia in the exposed fishes. Moreover, a significant decline in the serum immune indices such as LYZ, NO, and total IgM levels was also noticed. Tl exposure induced upregulation of IL-1β, TNF-α, and INF-γ genes in liver tissues, which are cytokines with pivotal immune functions in the fishes. Additionally, Tl increased the CMR (%) of Nile tilapia after challenge with pathogenic *A. hydrophila*. Collectively, these results draw the overall adverse impacts of sublethal Tl toxicity on fishes' immunity and disease resistance. Oppositely, the immunomodulatory results of AS positively ameliorated the negative impacts of Tl on the exposed fishes. At the same time, the valuable effects of diets supplemented with AS and their effects against the immunosuppressive effects of sub-lethal Tl toxicity partially diminished Tl toxicity compared to the control values. By this way, for better awareness about the mechanisms by which AS could mitigate Tl toxicity in fishes, additional studies are recommended.

## Data Availability Statement

The authors acknowledge that the data presented in this study must be deposited and made publicly available in an acceptable repository, prior to publication. Frontiers cannot accept a article that does not adhere to our open data policies.

## Ethics Statement

The animal study was reviewed and approved by all experiments in the current study have been done in line with the Local Experimental Animal Care Committee guidelines and approved by the Institutional Ethics Committee of Zagazig University, Egypt.

## Author Contributions

MF, MA, SK, WE, and EE-H contributed to the conception and design of the study. MF, MA, SK, and EE-H performed the experiments. MA and CM performed the statistical analyses. ADC, MA, and TI were involved in the project administration and funding acquisition. HA-L and MA wrote the first draft of the manuscript. All the authors have contributed to manuscript revision, read, and approved the submitted version of the manuscript.

## Funding

This study was funded by the Taif University Researchers Supporting Project number (TURSP-2020/134), Taif University, Taif, Saudi Arabia.

## Conflict of Interest

The authors declare that the research was conducted in the absence of any commercial or financial relationships that could be construed as a potential conflict of interest.

## Publisher's Note

All claims expressed in this article are solely those of the authors and do not necessarily represent those of their affiliated organizations, or those of the publisher, the editors and the reviewers. Any product that may be evaluated in this article, or claim that may be made by its manufacturer, is not guaranteed or endorsed by the publisher.
